# Disruption of estradiol regulation of orexin neurons: a novel mechanism in excessive ventilatory response to CO_2_ inhalation in a female rat model of panic disorder

**DOI:** 10.1038/s41398-020-01076-x

**Published:** 2020-11-10

**Authors:** Luana Tenorio-Lopes, Stéphanie Fournier, Mathilde S. Henry, Frédéric Bretzner, Richard Kinkead

**Affiliations:** 1 Hotchkiss Brain Institute; Department of Physiology and Pharmacology, University of Calgary, Calgary, AB Canada; 2 Centre de Recherche de l’Institut Universitaire de Cardiologie et de Pneumologie de Québec. Département de Pédiatrie. Université Laval, Québec, QC Canada; 3 INRAE, Université de Bordeaux, Bordeaux INP, Nutrineuro, UMR 1286, F-33000 Bordeaux, France; 4 Centre de Recherche du CHU de Québec-Université Laval, Axe Neurosciences. Département de Psychiatrie et de Neurosciences, Université Laval, Québec, QC Canada

**Keywords:** Physiology, Neuroscience

## Abstract

Panic disorder (PD) is ~2 times more frequent in women. An excessive ventilatory response to CO_2_ inhalation is more likely during the premenstrual phase. While ovarian hormones appear important in the pathophysiology of PD, their role remains poorly understood as female animals are rarely used in pre-clinical studies. Using neonatal maternal separation (NMS) to induce a “PD-like” respiratory phenotype, we tested the hypothesis that NMS disrupts hormonal regulation of the ventilatory response to CO_2_ in female rats. We then determined whether NMS attenuates the inhibitory actions of 17-β estradiol (E_2_) on orexin neurons (ORX). Pups were exposed to NMS (3 h/day; postnatal day 3–12). The ventilatory response to CO_2_-inhalation was tested before puberty, across the estrus cycle, and following ovariectomy. Plasma E_2_ and hypothalamic ORX_A_ were measured. The effect of an ORX_1_ antagonist (SB334867; 15 mg/kg) on the CO_2_ response was tested. Excitatory postsynaptic currents (EPSCs) were recorded from ORX neurons using whole-cell patch-clamp. NMS-related increase in the CO_2_ response was observed only when ovaries were functional; the largest ventilation was observed during proestrus. SB334867 blocked this effect. NMS augmented levels of ORX_A_ in hypothalamus extracts. EPSC frequency varied according to basal plasma E_2_ levels across the estrus cycle in controls but not NMS. NMS reproduces developmental and cyclic changes of respiratory manifestations of PD. NMS disrupts the inhibitory actions of E_2_ on the respiratory network. Impaired E_2_-related inhibition of ORX neurons during proestrus is a novel mechanism in respiratory manifestations of PD in females.

## Introduction

Many panic disorder (PD) patients experience respiratory symptoms, including hyperventilation, sleep apnea, chest pain, and dyspnea^1–3^. According to Klein’s “*False suffocation alarm hypothesis*”, excessive sensitivity to respiratory stimuli is at the core of PD^4^. CO_2_ inhalation can trigger intense fear, autonomic, and ventilatory responses^5^ and the probability of experiencing the panicogenic effects of CO_2_ is greater in PD patients than healthy subjects^6,7^. Located in a hypothalamic region initially known as the “panic area”, orexin-producing neurons (ORX) regulate arousal and the intensity of respiratory reflexes^8–10^. ORX concentration in the cerebrospinal fluid of PD patients is higher than in healthy subjects^11^ and activation of ORX neurons and ORX-1 receptors are both necessary to observe a panic-prone state in rats and in humans^11–13^.

Early life adversities are an important risk factor for PD; clinical and pre-clinical data show that unstable parental conditions or experimental disruption of maternal care augment respiratory and behavioral responses to CO_2_^14–16^. Much like chronic stress, neonatal maternal separation (NMS) augments ORX function^17–19^. Together, these data strongly argue that NMS-related dysregulation of ORX function is an important mechanism in excessive ventilatory response to CO_2_ inhalation in PD patients. The mechanisms responsible for abnormal ORX-modulation of the ventilatory control system are not fully understood, but to make significant progress that will ultimately influence clinical practice, pre-clinical research requires animal models that are close to the clinical reality^20,21^.

The prevalence of PD is 2–3 fold greater in women than in men, yet most of our basic knowledge arises from experiments performed on males despite strong evidence indicating that endocrine regulation of ORX neurons is sex-specific^22,23^. This is an important issue because clinical observations point to an important role of ovarian hormones in the pathophysiology of PD. The incidence of PD rises at puberty and in adult women, the responsiveness to CO_2_ inhalation is highest during the pre-menstrual phase. Together, these observations suggest that PD patients are more sensitive to hormonal fluctuation than healthy subjects^24–26^. To address this issue, we first tested the hypothesis that early life adversities (in the form of NMS) disrupts hormonal regulation of the ventilatory response to CO_2_. Specifically, we determined whether NMS-related increase in the responsiveness to CO_2_ inhalation evolves with reproductive status of females. Non-invasive respiratory measurements were performed prior to puberty, across each phase of the estrus cycle, and following ovariectomy. We then used a pharmacological approach to determine whether activation of ORX_1_-receptors is necessary to NMS-induced enhancement of the CO_2_ response. The evidence indicating that 17β-estradiol (E_2_) inhibits ORX neurons being indirect^27,28^, we then used whole-cell patch-clamp recordings to assess E_2_’s effects on green fluorescent protein (GFP)-labeled ORX neurons in females. Finally, we tested the hypothesis that NMS disrupts E_2_’s inhibitory actions on the ORX system and its influence on CO_2_-induced respiratory manifestation of PD in females.

## Methods

### Animals and ethical approval

Experiments were performed on sexually mature female Sprague–Dawley rats and pre-pubertal rat pups (14–15 days old) of both sexes. Rats were preferred to mice because, unlike mice, enhancement of the CO_2_ response due to early life adversities is sex-specific in this species^15,29^. Details about age, body weight, and animal distribution amongst the experimental groups are reported in Supplemental Table 1 and in the figures. All animals were born and raised in our animal care facilities. Rats were supplied with food and water *ad libitum* and maintained in standard conditions (21 °C, 12:12-h dark–light cycle: lights on at 06:00 and off at 18:00). Animal Care Committee of Université Laval approved all the experimental procedures and protocols, which were in accordance with the guidelines of the Canadian Council on Animal Care.

### Neonatal maternal separation (NMS)

The NMS protocol was identical to the one used in our previous studies^29,30^. Briefly, virgin females were mated and 3 days after delivery, each litter was separated daily from their mother 3 h/day (09:00–12:00) from postnatal day 3–12. Control animals were undisturbed during the same period; see the Supplement for details. Rats were weaned and raised under standard animal care conditions until experiments were performed. For each group, rats originated from multiple litters to avoid litter-specific effects.

### Whole-body plethysmography and tissue sampling

Ventilatory variables were measured in unrestrained, unanesthetized rats using whole-body plethysmography according to standard procedures^30^. 45 to 60 min prior to the recording, females were injected either with vehicle or with the selective ORX_1_ receptor antagonist (SB334867; 15 mg/kg). Ventilation was recorded at rest (room air) followed by hypercapnic exposure (5% CO_2_, balance air; 10 min). CO_2_ levels used to assess ventilatory and behavioral responsiveness varies between 5 and 35% in animals and humans^31,32^. The level chosen here ensured a robust ventilatory response with minimal change in behavior to avoid movement artefacts that interfere with respiratory measurements. At the end of the experiment, rats were deeply anesthetised; as NMS increases vaginal sensitivity^33^, determination of the estrus cycle by vaginal smear was performed only at this time to avoid influencing results. Blood and brains were then harvested to obtain post-CO_2_ samples immunohistochemistry and quantification of plasma E_2_ levels. Experiments were performed between 13:00 and 15:00. Note that blood and brains were also obtained from a distinct group exposed to room air to obtain baseline data for E_2_ and quantification of ORX_A_ in hypothalamus extracts. For setup, protocol, and data collection details, see the Supplement.

### Ovariectomy and 17β-estradiol (E_2_) replacement

Ovariectomy (OVX) was used to reduce circulating ovarian hormones chronically; surgery was performed according to standard procedures^34^. Two weeks after surgery, the CO_2_ response was measured and compared between NMS and controls. A distinct group of OVX females received either vehicle (peanut oil; 100 µl) or one E_2_ injection (3 or 10 µg) every 4 days to restore E_2_ level within physiological range and mimic cyclic fluctuations. The effects of higher E_2_ supplementation was tested in another group of OVX females by injecting with 25 µg. The last injections were performed on the day of the experiments. Based on the evidence suggesting that E_2_ inhibits ORX neurons^27,28^, only E_2_ was used in the replacement.

### *c-Fos*/orexin-A immunohistochemistry

We first used *c-Fos* protein expression to determine whether NMS augments neuronal activation of ORX neurons in females. Based on ventilatory measurements, this initial evaluation was performed during the proestrus phase only. 40 μm coronal brain sections were double-labeled with primary antibodies against *c-Fos* and ORX_A_ to confirm cell phenotype. Since the physiological function of ORX neurons differs between hypothalamic sub regions^35–37^, single (ORX_A_ only) and double-labeled cells were counted in the dorsomedial and lateral hypothalamus (DMH and LH, respectively) and the perifornical area (PeF). See Supplement for details.

### Whole-cell patch-clamp recording of orexin neurons

#### Identification of orexin neurons with an adeno-associated virus (AAV)

Four weeks-old females were injected with an AAV construct that expresses a green fluorescent protein (GFP) under the control of an ORX promoter. During surgery, rats received unilateral injections (1 µl/side) of the ORX:GFP virus in the following stereotaxic coordinates (from Bregma: RC: −2.6 mm; ML: 1.2 mm; DV: −9.0 mm). A 4-week recovery period ensured consistent GFP labeling; the intensity of GFP labeling observed in the PeF area was more apparent than in adjacent areas.

#### Slice electrophysiology

Hypothalamic slices (300 µm) containing GFP-labeled ORX neurons were used for whole-cell patch-clamp recording of basic electrophysiological properties, excitatory postsynaptic currents converging onto ORX neurons, and responses to E_2_ application (100 nM; 10 min). E_2_ concentration was based on the literature^38^. Slice preparation and recording procedures were performed as described previously^39,40^. Since ORX neurons of the LH do not contribute to cardiorespiratory regulation^37^, recordings were performed in the PeF/DMH. The estrus cycle was determined after the brain was harvested; data were compared between NMS and controls. See the Supplement for details.

### Statistics

Multifactorial analysis of variance (ANOVA) assessed the effects of NMS and estrous cycle on respiratory variables, 17β-estradiol (E_2_), immunohistochemical, and electrophysiological data. CO_2_ exposure was also considered for analyses of respiratory data and E_2_; a repeated measures design was used when appropriate; equality of variance was tested. The relationship between E_2_ and the ventilatory response to CO_2_ was assessed using analysis of co-variance (ANCOVA) and a correlation *z*-test. Since patch-clamp data often originate from multiple cells from the same animal, a mixed ANOVA model (mixed-effect model) was used to ensure that between-group differences were not attributable to a specific subject^41^. ANOVA results are reported in the figures for clarity and conciseness. All data are presented as means ± SEM. A significant ANOVA results (*P* ≤ 0.05) was followed by a *post hoc* test (Fisher’s least significant difference) to identify specific differences; a Bonferroni correction was applied when multiple comparisons were performed. Analyses were performed using Statview 5.0 (SAS Institute, Cary, NC, USA) and *JASP* (version 0.13; University of the Netherlands).

## Results

### The ventilatory response to CO_2_ and 17β-estradiol (E_2_) levels across the female’s reproductive status

**Fig. 1:**
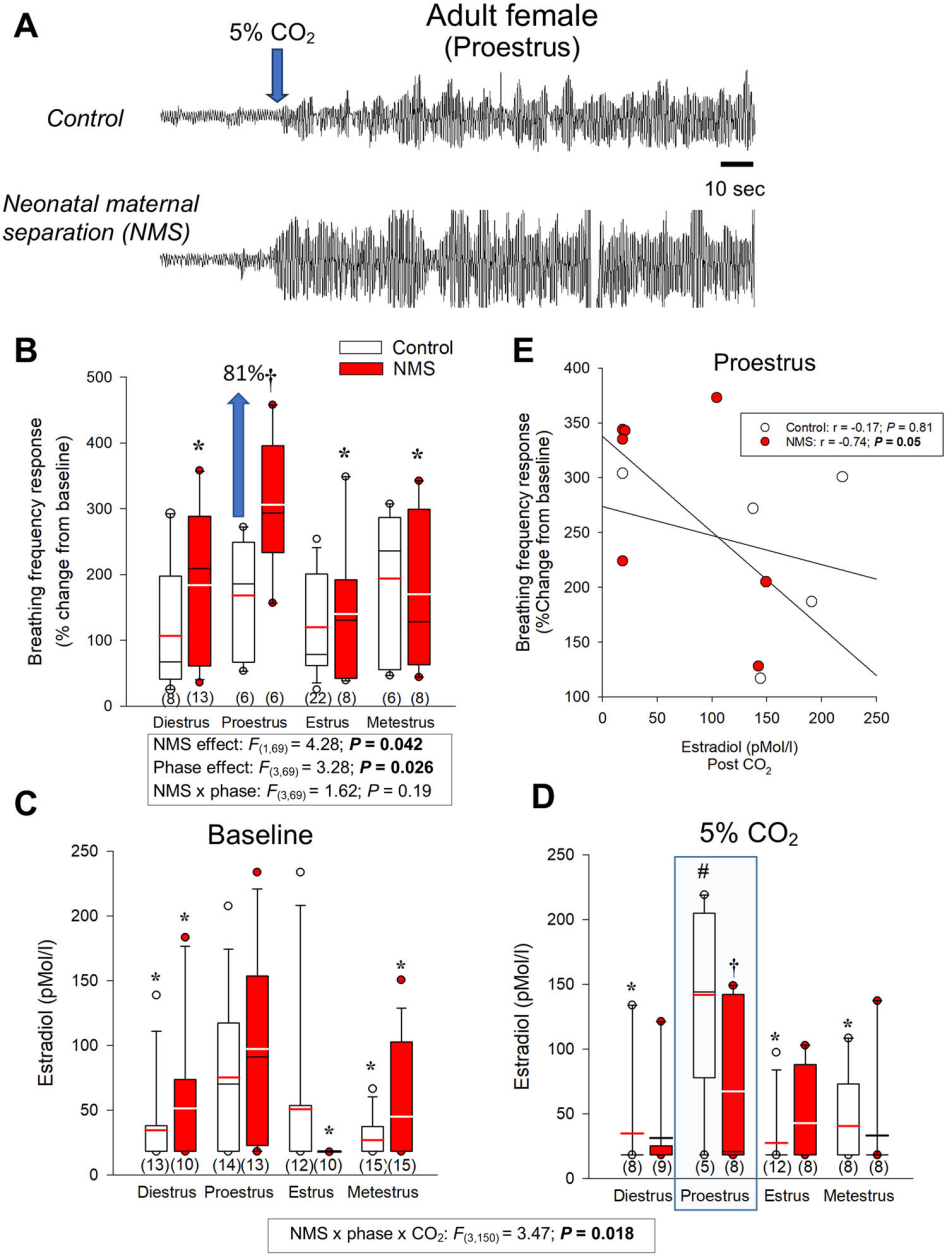
Comparison of the hyperventilatory response to CO_2_ inhalation (5% CO_2_; 10 min) between adult females raised under standard conditions or subjected to neonatal maternal separation (NMS) across the different phase of the estrus cycle. **A** Original plethysmographic recording comparing ventilatory activity at rest and at the onset of CO_2_ inhalation (blue arrow) in a female raised under control conditions (top trace) versus a female subjected to neonatal maternal separation (bottom trace; NMS: 3 h/day; postnatal days 3–12). **B** The breathing frequency response expressed as a percentage change from baseline (room air). 17β-estradiol (E_2_) levels across the estrus cycles measured **C** while breathing room air (baseline) and **D** 30 min following CO_2_ inhalation test (5% CO_2_; 10 min). **E** Regression analysis comparing the relationship between E_2_ levels during proestrus (post-CO_2_; blue box) and the intensity of the breathing frequency response to CO_2_ between NMS (red circles) and control females (white circles). Box plots: the top and bottom boundaries of the box indicate the 25^th^ and 75^th^ percentile, respectively. Within the box, the black bar indicates the median; the other bar (red or white) indicate the mean. The error bars indicate the 10^th^ and 90^th^ percentiles. The numbers in brackets below the boxes indicate the number of replicates in each group. *Post hoc* pairwise comparisons were performed only when warranted by ANOVA. *Indicates a value different from corresponding proestrus value at *P* < 0.05. ^†^Indicates a value different from corresponding control value at *P* < 0.05.

CO_2_ inhalation induced a rapid and robust hyperpnoea, which was greater in NMS females than controls (Fig. 1A, B). The intensity of the respiratory frequency and minute ventilation responses varied across the different phases of the estrus cycle and the largest difference between NMS and controls were observed during proestrus (Fig. 1A, B and Supplementary Fig. 1B). Baseline E_2_ levels fluctuated across the estrus cycle and peaked during proestrus; NMS did not affect those values (Fig. 1C). During proestrus, CO_2_ exposure augmented E_2_ levels in control, but not NMS females (Fig. 1D). The E_2_ levels measured following CO_2_ exposure during proestrus were inversely correlated with the intensity of the breathing frequency response in NMS but not controls (Fig. 1E).

**Fig. 2:**
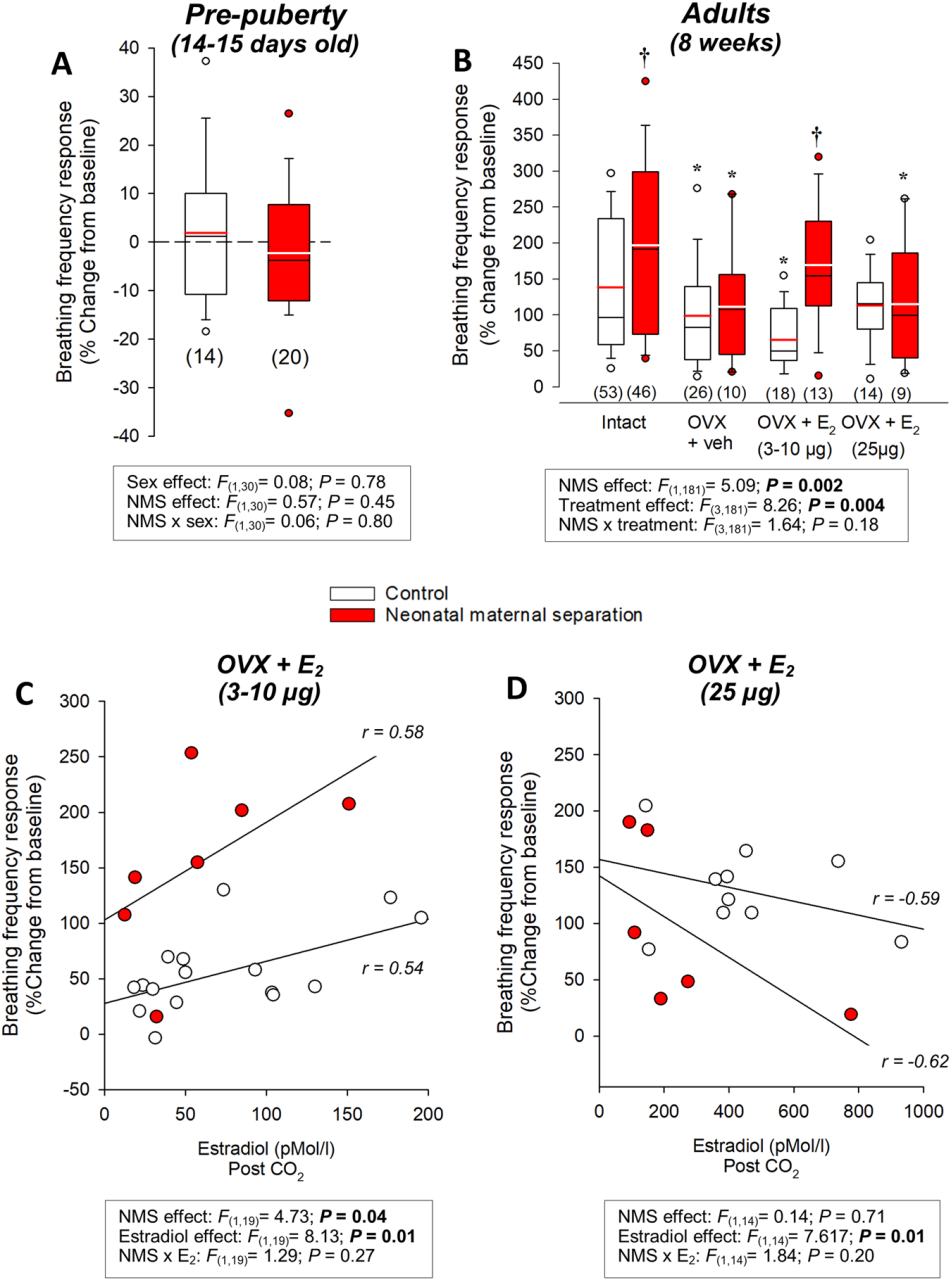
Importance of ovarian function on the breathing frequency response to CO_2_ inhalation (5% CO_2_; 10 min). Breathing frequency response to CO_2_ inhalation (5% CO_2_; 10 min) in **A** pre-pubertal rat pups (14–15 days old) and **B** adult females with intact ovaries or following (OVX) with and without 17-β estradiol (E_2_) replacement. Data from intact adult females include females without surgical procedure (from Fig. 1) and females that subjected to sham surgery that received vehicle injections (peanut oil; 100 µl). Ovariectomy or sham surgeries were performed 2 weeks prior to ventilatory measurements. E_2_ replacement reproduced cyclic fluctuations by performing a daily injection every 4 days over 12 days prior to the experiments (4 injections in total). Each injection contained either vehicle (peanut oil, 100 µl) or E_2_ (3 or 10 µg; normal levels or 25 µg; high levels). The histograms represent the frequency responses expressed as a percentage change from baseline (room air). Data are compared between rats raised under standard conditions (white bars) or subjected to neonatal maternal separation (NMS; red bars). Box plots: the top and bottom boundaries of the box indicate the 25^th^ and 75^th^ percentile, respectively. Within the box, the black bar indicates the median; the other bar (red or white) indicate the mean. The error bars indicate the 10^th^ and 90^th^ percentiles. The numbers in brackets below the boxes indicate the number of replicates in each group. *Post hoc* pairwise comparisons were performed only when warranted by ANOVA. *Indicates a value different from the corresponding intact value at *P* < 0.05. ^†^Indicates a value different from the corresponding control value at *P* < 0.05. The relationship between plasma E_2_ levels and the intensity of the hyperventilatory response in OVX females supplemented with **C** normal E_2_ (3–10 µg) or **D** high E_2_ (25 µg).

**Fig. 3:**
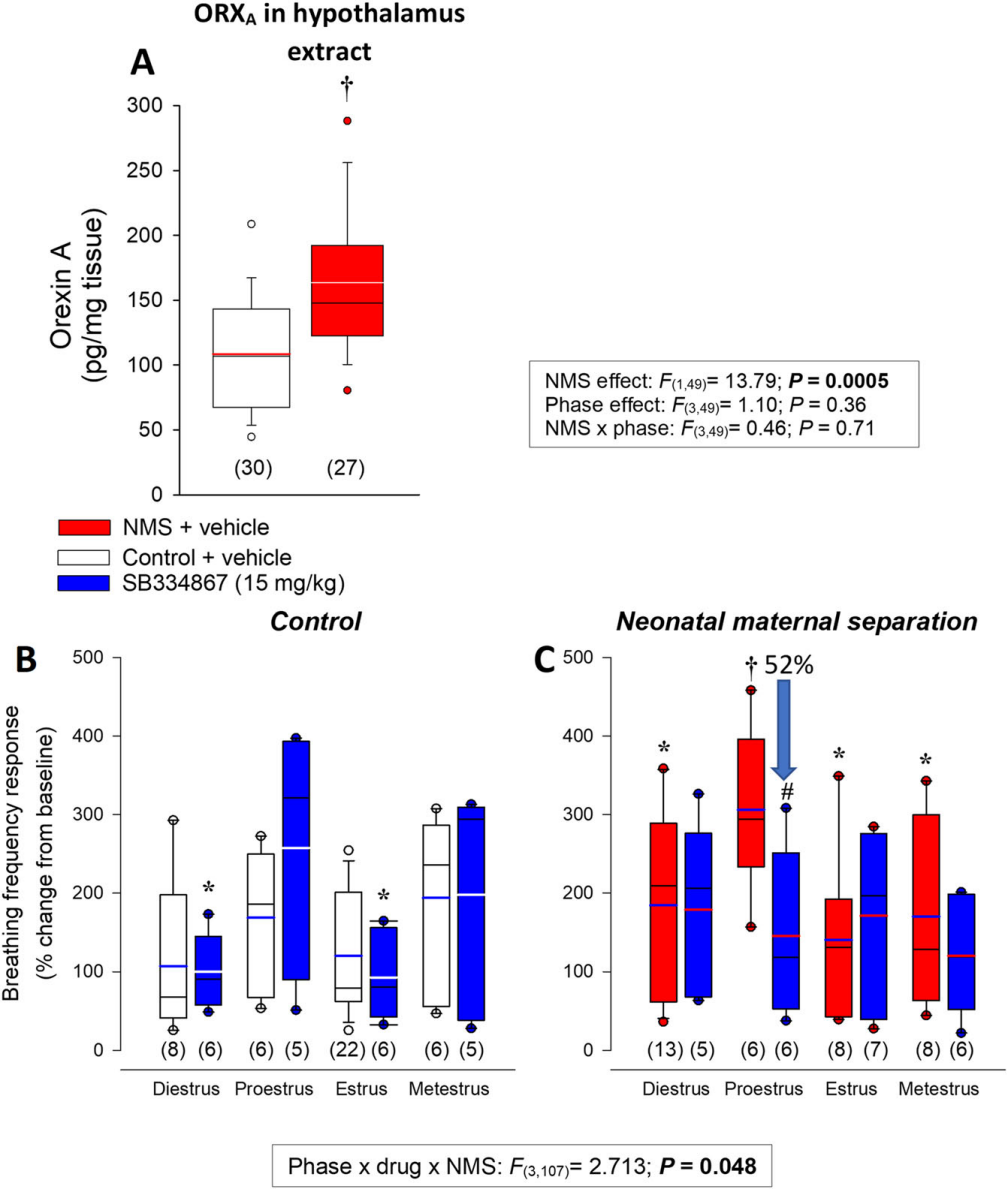
Effects of neonatal stress on ORX_A_ in the hypothalamus and effectiveness to the selective orexin-1 receptor antagonist SB334867 on the hyperventilatory response to CO_2_. **A** Comparison of basal ORX_A_ levels in hypothalamus extracts from females subjected to neonatal maternal separation (NMS; red bars) or raised under control conditions (white bars). Data were obtained from different each phase of the estrus cycle; however, data are pooled since the effect of ovarian status was not significant. **B** Effect of pre-treatment with SB334867 on the breathing frequency response of control females and **C** females subjected to NMS. Data were obtained during each phase of the estrus cycle; breathing was measured at the end of the CO_2_ inhalation test (5% CO_2_; 10 min) and expressed as a percentage change from baseline (room air). Data from SB334867-treated rats (15 mg/kg; I.P.; blue bars) are compared with those that received an injection of vehicle (50 mM HP-β-CD—10% + DMSO—2%) 45–60 min before start the plethysmography recordings. Box plots: the top and bottom boundaries of the box indicate the 25^th^ and 75^th^ percentile, respectively. Within the box, the black bar indicates the median; the other bar (red, white, or blue) indicate the mean. The error bars indicate the 10^th^ and 90^th^ percentiles. The numbers in brackets below the boxes indicate the number of replicates in each group. *Post hoc* pairwise comparisons were performed only when warranted by ANOVA. *Indicates a value statistically different from corresponding proestrus value at *P* < 0.05. ^†^Indicates a value different from corresponding control value at *P* < 0.05. ^#^Indicates a value different from corresponding vehicle value at *P* < 0.05.

Prior to puberty, the hypercapnic ventilatory response was modest^42^; the response did not differ between sexes and was unaffected by NMS (Fig. 2A and see Supplementary Fig. 1C, D for effects on other respiratory variables). In adults, OVX reduced E_2_ levels (Supplementary Fig. 2) and the CO_2_ response such that the intensity of the hyperpnoea was now similar between groups (Fig. 2B). In OVX females, the first E_2_ supplementation protocol restored plasma E_2_ to physiological levels (Fig. 3C) and increased the respiratory frequency response in NMS but not controls. In both groups, the response was directly proportional to E_2_ levels (Fig. 2B, C and Supplementary Fig. 1). While the E_2_ levels achieved with the second supplementation protocol exceed normal values (Fig. 2D), this treatment demonstrated that at that elevated E_2_ inhibits the ventilatory response to CO_2_ (Fig. 2D and Supplementary Fig. 1E, F).

None of the ventilatory variables and other indicators of metabolism measured at rest differed between groups. Breathing frequency of “resting” OVX females was slightly higher than intact females but this was not sufficient to augment minute ventilation (Supplementary Table 1).

### Excessive ORX modulation contributes to NMS-related increase in CO_2_ response

**Fig. 4:**
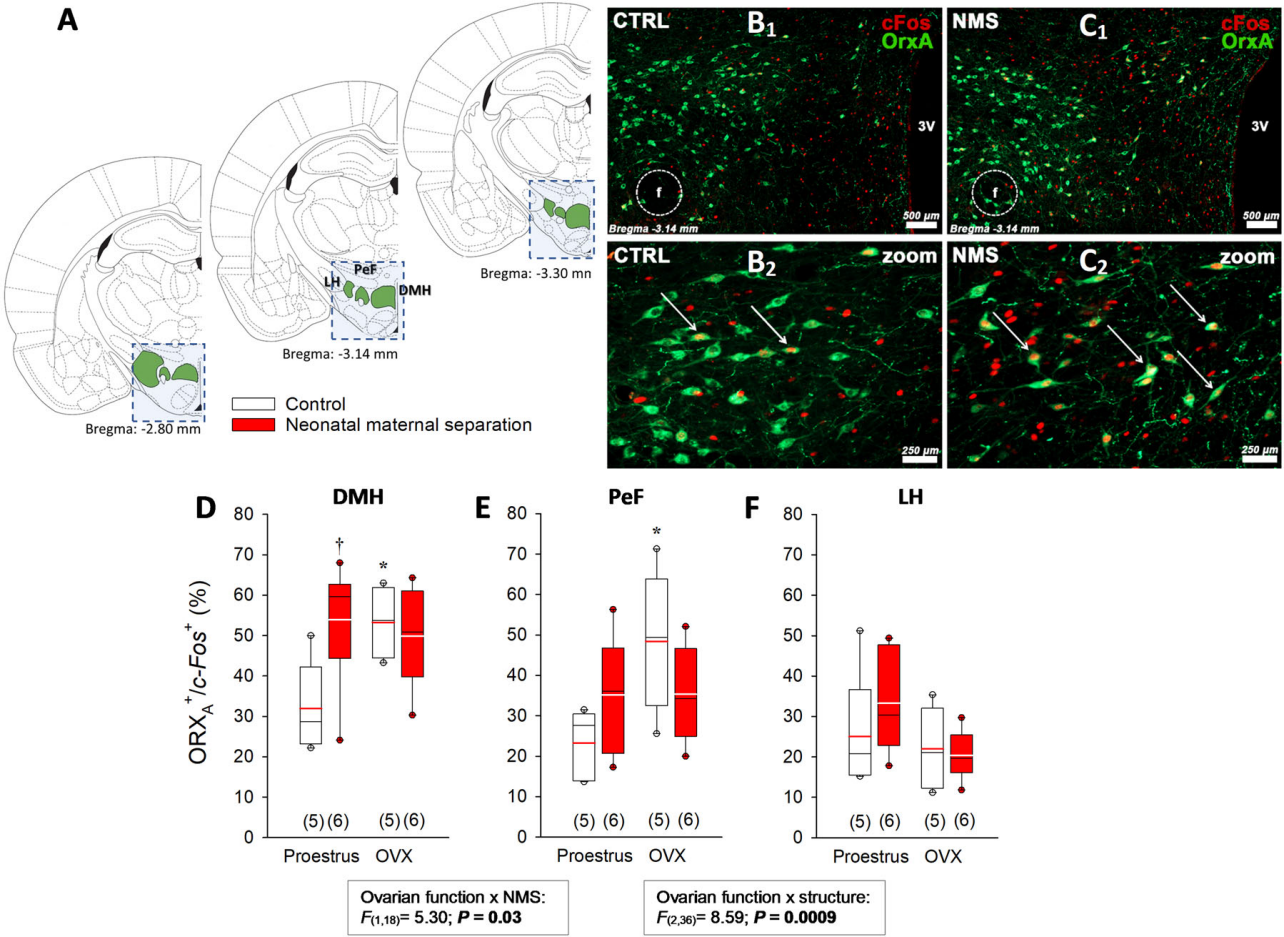
Neonatal maternal separation (NMS) augments hypothalamic levels of orexin A (ORX_A_) and augments *c-Fos* immunolabelling in ORX_A_ neurons in adult female rats. **A** Schematic representation of frontal brain sections illustrating the hypothalamic region (blue square) that was isolated and homogenised to measure basal ORX_A_ levels by ELISA assay. The schematic also illustrates the regions containing ORX neurons that were delineated to quantify *c-Fos* immunolabelling (adapted with permission from ref. ^72^). Photomicrographs comparing *c-Fos* (Texas red) and ORX_A_ (FITC) immunolabelling between tissue sections from females in proestrus **B**_**1**_ raised under control conditions (CTRL) or **C**_**2**_ previously subjected to NMS (3 h/day, postnatal days 3–12). The third ventricle (3 V) and fornix (f) are identified as landmarks. Panels **B**_**2**_ and **C**_**2**_ were obtained at higher magnification for CTRL and NMS, respectively and white arrows identify double-labeled neurons. **D** Comparison of the proportion of ORX_A_ positive neurons expressing *c-Fos* in the dorsomedial hypothalamus (DMH) between NMS and controls during proestrus and two weeks following ovariectomy (OVX). Similar analyses were performed in **E** the perifornical area (PeF) and **F** the lateral hypothalamus. Box plots: the top and bottom boundaries of the box indicate the 25^th^ and 75^th^ percentile, respectively. Within the box, the black bar indicates the median; the other bar (red or white) indicate the mean. The error bars indicate the 10^th^ and 90^th^ percentiles. The numbers in brackets below the boxes indicate the number of replicates in each group. *Post hoc* pairwise comparisons were performed only when warranted by ANOVA. ^†^Significantly different from corresponding control value at *P* ≤ 0.05. *Indicates a value statistically different from corresponding proestrus value at *P* < 0.05.

Anomalies in ORX neurotransmission contributes to the pathophysiology of a panic-prone state in rats and in humans^11–13^. To determine whether enhanced ORX contributes to NMS-related increase in CO_2_ responsiveness in females, we first quantified ORX_A_ levels in hypothalamus extracts; values obtained in NMS females were 51% higher than controls (Fig. 3A). We then inactivated ORX_1_ receptors by pre-treatment with SB334867. The treatment did not affect breathing at rest (Supplementary Table 1) and generally had limited effects on the CO_2_ response; however, SB334867 prevented the excessive ventilatory response of NMS rats during proestrus (Fig. 3C and Supplementary Fig. 3). As physiological data indicate that NMS-related anomalies in respiratory control are more important during proestrus (Fig. 1), we used *c-Fos* immunolabeling to determine if ORX neurons of NMS females were more active than control during that phase (Fig. 4). By comparing data with OVX females, we evaluated the sensitivity to E_2_ withdrawal between groups. In the DMH, the percentage of ORX neurons expressing *c-Fos* was greater in NMS than control during proestrus. Since OVX increased this ratio only in controls, the level achieved was now similar between groups (Fig. 4D). While a similar trend was observed in the PeF, neither NMS nor OVX affected the number of double-labeled cells in the LH (Fig. 4E and F, respectively).

### Neonatal maternal separation disrupts E_2_ regulation of orexin neurons of the PeF/DMH

**Fig. 5:**
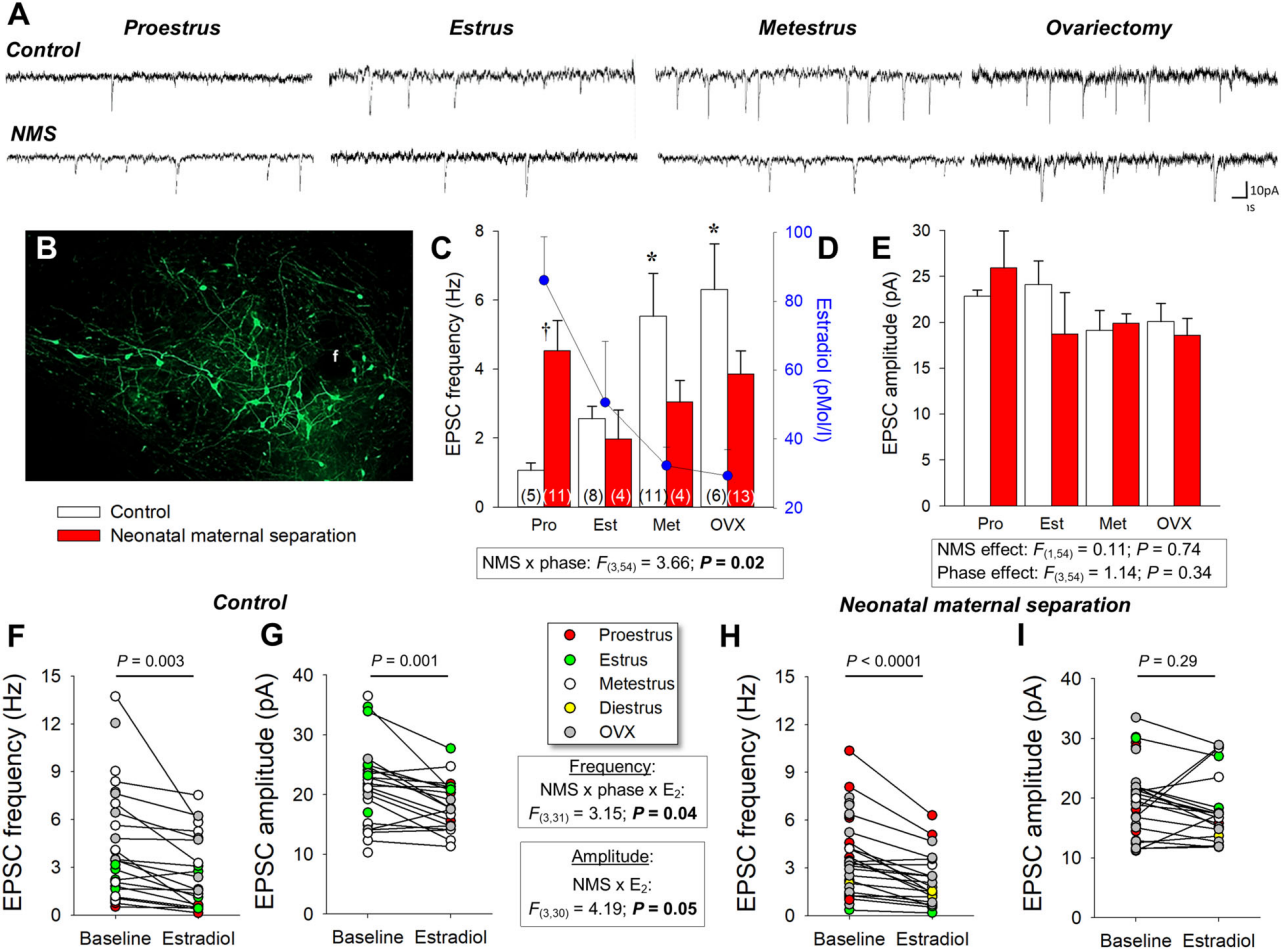
Neonatal maternal separation (NMS) reduces the excitatory postsynaptic currents (EPSC) recorded in GFP-labeled orexin neurons in response to natural and ovariectomy-induced changes in 17β-estradiol (E_2_) level. **A** Comparison of EPSC recordings from orexin neurons between cells obtained from females during different phase of the estrus cycle and 2 weeks following ovariectomy (OVX); tissue slices originated from females raised under control conditions (top traces) or subjected to neonatal maternal separation (NMS, bottom traces; 3 h/day, postnatal days 3–12). **B** Photomicrograph illustrating GFP-labeled orexin neurons; the fornix (f) is shown as a landmark. **C** Population data of EPSC frequencies recorded during three distinct phases of the estrus cycle and following OVX. **D** Baseline E_2_ values from Fig. 2A measured in each stage are reported for comparison; values from NMS and controls were pooled since they are not statistically different. **E** Reports EPSC amplitudes. Note that since recordings in diestrus were performed only in controls (see **F**, **G**), data for this stage could not be compared between groups. Effect of E_2_ application (10 min; 100 nM) on EPSC **F** frequency and **G** amplitude in slices from control females. EPSC frequency and amplitude data from NMS females are reported in (**H**, **I**), respectively. Within histogram bars, the numbers in brackets indicate the number of replicates in each group. Data are expressed as the mean ± SEM. *Post hoc* pairwise comparisons were performed only when warranted by ANOVA. ^†^Significantly different from corresponding control value at *P* ≤ 0.05. *Indicates a value statistically different from corresponding proestrus value at *P* < 0.05.

As our data indicate that NMS-related potentiation of ORX action on respiratory control is the greatest during proestrus, we then used whole-cell patch-clamp recording to assess the impacts of NMS and E_2_ on ORX neurons. In naturally cycling females, membrane potential (V*m*) did not change across the estrus cycle and values recorded in NMS females were slightly lower than controls. However, OVX augmented cell capacitance and the resting V*m* of ORX neurons in NMS but not controls (Supplementary Table 2). None of the other basic cell properties was influenced by NMS or ovarian function (Supplementary Table 2). Ovarian status influenced the excitatory postsynaptic currents (EPSC) frequency, especially in controls (Fig. 5A, C). In those females, EPSC frequency was inversely proportional to the basal level of E_2_ associated with each phase of the estrus cycle (Fig. 5C). In contrast with controls, ORX neurons from NMS females had the highest EPSC frequency during proestrus, when E_2_ levels peaked (Fig. 5A, C). EPSC amplitude was not affected by NMS or the estrus cycle (Fig. 5A, E). Bath application of E_2_ reduced EPSCs frequency in both control and NMS; however, the largest drop was observed during proestrus in NMS females (−0.63 versus −4.09 Hz for control and NMS, respectively). E_2_ reduced EPSC amplitude in ORX cells from control but not NMS (Fig. 5F, G–I).

## Discussion

Puberty and cyclic fluctuations in ovarian hormones are normal physiological processes but in a subpopulation of women, these events contribute to the onset and cyclic exacerbation of PD^43–45^. Thus, elucidating how ovarian function affects respiratory manifestations of PD is of utmost importance to our understanding of the pathophysiology of this disorder. Early life adversities are a significant risk factor for PD and the sex-specific enhancement of the ventilatory response to CO_2_ inhalation in rats is an attractive model to address this question^29^. Here, we show that in NMS females endogenous release of E_2_ during CO_2_ inhalation is insufficient to maintain the ventilatory response within a normal range. These data support our main hypothesis and indicate that mature (functional) ovaries are necessary to observe an excessive ventilatory response to CO_2_ inhalation in NMS females. This, and the involvement of the ORX system in this process strengthens the model and our demonstration that NMS disrupts E_2_ regulation of ORX neurons point to a novel mechanism in the pathophysiology of PD in females.

### Neonatal maternal separation affects E_2_ signaling regulating the response to CO_2_ inhalation

Progesterone and E_2_ both fluctuate across the estrus cycle. However, the largest NMS-related increase in CO_2_ response coincided with the peak in E_2_ (proestrus) and following OVX, restoring normal E_2_ levels alone was sufficient to reinstate an excessive ventilatory response in NMS rats. We, therefore, conclude that E_2_ plays a primary role in the enhanced response to CO_2_. NMS does not affect the cyclic changes in E_2_ or progesterone under basal conditions (Fig. 1C;^46^) but attenuates E_2_ release following CO_2_ exposure, especially during proestrus. The sympathetic system regulates ovarian E_2_ secretion^47^, and ovarian aromatase expression peaks during proestrus^48^. Since plasma E_2_ closely reflects brain levels^49^, such impairment in E_2_ signalling within the brain of NMS females is likely. The inability of NMS females to augment E_2_ in response to CO_2_ during proestrus suggests that NMS reduced E_2_ synthesis capacity and thus compromise the response to an acute challenge. The relationships between E_2_ levels and the intensity of the ventilatory response suggest that impairment of E_2_ signalling contributes to the abnormal respiratory phenotype of NMS females. Experiments performed on OVX females show that E_2_ can attenuate the excessive CO_2_ response of NMS females; however, higher levels are necessary.

### Neonatal maternal separation augments ORX activation under basal conditions

Having previously shown that NMS does not affect the carotid body’s response to CO_2_ in male and female rats^50^, our investigation focused on central mechanisms regulating the ventilatory response to CO_2_. Orexin neurons project to key medullary areas regulating breathing, including those that generate respiratory rhythm and contribute to CO_2_ chemosensitivity^9^. The clinical evidence implicating ORX in PD is important^11,51^ and our results showing that NMS augments ORX_A_ levels in the hypothalamus are consistent with clinical and preclinical data. A similar increase has been reported in males^18^ but since regulation of ORX neurons likely differs between sexes^19,22^, testing this effect in females was necessary. ORX synthesis is activity-dependent and highly plastic^52^. In light of the close relationship between the hypothalamo-pituitary adrenal (HPA) axis and the ORX system^19,53^ the enhancement of basal HPA activity commonly reported in animals and humans who experienced early life adversities could explain the higher ORX_A_ level in hypothalamic extracts^54–56^. In adult rats, however, disruption of HPA axis function by NMS is significant only in males^57,58^. Thus, another mechanism should be considered to explain this result.

In control females, comparison of *c-Fos* expression between females experiencing high (proestrus) and low (OVX) E_2_ clearly supports an inhibitory action of estrogens on ORX neurons. Conversely, the high *c-Fos*/ORX_A_ ratio observed in NMS females, regardless of the ovarian function, suggests a generally higher degree of basal activity and a reduced sensitivity to E_2_ and/or insufficient levels. This result therefore provides a plausible explanation for greater level of ORX_A_ in hypothalamic extracts and the larger ventilatory response to CO_2_ inhalation. The latter interpretation is supported by fact that NMS augmented the *c-Fos*/ORX_A_ ratio in PeF/DMH areas that, unlike the LH, regulate cardiorespiratory homeostasis^37^. Moreover, pre-treatment with SB334867 prevented NMS-related increase in the ventilatory response to CO_2_.

### Neonatal maternal separation disrupts E_2_ regulation of ORX neurons

ORX neurons are essential to several homeostatic functions. To the best of our knowledge, this is the first study documenting the impact of natural fluctuations in ovarian hormones (and OVX) on basic properties and excitatory synaptic inputs in females. While natural fluctuations in ovarian hormones have no impact on basic properties, the V*m* and capacitance values obtained during the natural E_2_ nadir (metestrus) differs from those recorded following OVX. While OVX is the gold standard in preclinical research for evaluating gonadal hormone effects in females^59^, these results remind us that the changes induced by OVX may be more complex than a simple reduction of circulating hormones. Keeping that limitation in mind, the opposing effects of OVX on V*m* between NMS and controls nonetheless indicate that NMS affects the way ovarian hormones influence this important property of ORX neurons.

Orexin neurons are the target of multiple afferent signals form diverse origins^10,60,61^. The frequency and amplitude of spontaneous EPSC’s reflect the number and the strength of excitatory synaptic inputs acting on ORX neurons, respectively. E_2_ acts via both membrane and nuclear receptors and the results reported here provide valuable insights into the mechanisms by which endogenous E_2_ contributes to inhibition of ORX neurons. EPSC frequencies measured in controls were inversely related to basal plasma E_2_ levels associated with natural cyclic fluctuations or OVX; exogenous E_2_ elicited a similar decrease in frequency in the minutes that followed its application onto slices. This implies that both E_2_ receptor types could regulate the number of synapses converging onto ORX neurons. Conversely, the fact that only acute E_2_ reduced EPSCs amplitude suggests that regulation of synaptic strength by E_2_ signalling is rapid but transient.

E_2_ is generally known to promote dendritic spine formation, potentiate excitatory synaptic transmission, and reduce the efficacy of GABAergic inhibition^62–65^. In the cortex and the hippocampus, however, application of LY 3201 (a selective agonist of the nuclear receptor ERβ) can elicit an opposite response by reducing dendritic spines and increasing expression of glutamic acid decarboxylase (GAD)^66^. This increase in GAD expression, combined with a reduction in the expression of NMDA receptors shifts the balance between excitatory and inhibitory neurotransmission in favor of inhibition^66^. Together, these effects explain the anxiogenic and anxiolytic actions of ERα and ERβ, respectively^67^. Since E_2_ inhibits expression of ERs^68^, region-specific changes in the relative expression of ERα and ERβ likely contribute to the phase-dependent effects reported here. While NMS affects ERβ expression in the hippocampus of males^69^, its impact in females is yet to be tested.

At the system level, the CO_2_ responses measured in controls indicate that as E_2_ declines across the cycle, the increased activation of ORX cells is dampened to prevent excessive hyperpnoea. Obviously, this mechanism is not fully functional in NMS females. Regulation of ORX neurons is a complex process that involves an important local network of neurons and astrocytes^60^ but obviously, NMS reduces E_2_’s actions on these cells. In fact, the CO_2_ response following E_2_ replacement in OVX NMS females suggests that depending on the concentration administered, E_2_ may have excitatory or inhibitory effects. Interestingly, PD is rare following menopause but E_2_ replacement therapy has been linked with the development of panic attacks in some patients^70^.

## Limitations and conclusion

Inadequate modeling of human disease hinders translation of basic knowledge into effective treatment for human^21^. While our study shows that NMS closely reproduces developmental and cyclic changes in the respiratory manifestations of PD and enhancement of ORX modulation, we must keep in mind that animal research cannot reproduce the complex psychosocial reality often associated with PD. Furthermore, the estrus cycle in rodents is not equivalent to the menstrual cycle in humans^59^. That being said, NMS nonetheless meets key criteria expected from an animal model, including time-dependent and sex-specific effects on respiration^20,71^. The results reported here, therefore, offer valuable insights into the basic mechanism in this neurological disorder affecting female rats. Our demonstration that NMS disrupts the inhibitory actions of E_2_ on respiratory control are significant as they offers new avenues to alleviate PD.

## Supplementary information

Supplementary methods

Supplementary Table 1

Supplementary Table 2

Supplementary Figure 1

Supplementary Figure 2

Supplementary Figure 3
