# Supplementary material for: Disruption of estradiol regulation of orexin neurons: a novel mechanism in excessive ventilatory response to CO2 inhalation in a female rat model of panic disorder
Source: Transl Psychiatry. 2020 Nov 10;10:394. doi: 10.1038/s41398-020-01076-x (PMC7656265; doi:10.1038/s41398-020-01076-x)
Supplement: Supplementary file 1 — Supplementary methods [file 41398_2020_1076_MOESM1_ESM.docx]

***Supplement***

**MATERIALS AND METHODS**

Note that unless specified otherwise, all chemicals were purchased from Sigma-Aldrich, Canada.

**Mating and neonatal maternal separation (NMS) procedures**

Virgin females were mated and delivered 12-15 pups. Two days after delivery, litters were culled to 12 pups, when necessary. After removing the mother from the home cage, the separated pups were placed in a temperature (35°C) and humidity (45%) controlled incubator and isolated from each other by a cardboard partition. The mother remained away from the home cage for the entire NMS protocol. Mother and pups were reunited in the home cage at the end of the separation procedure. Control animals (CTRL) were continuously maintained under standard animal care procedures throughout the neonatal period with minimal human contact. Since an undisturbed cage avoids all protential effects of animal manipulation on maternal behavior, these animals are the most desirable reference group for investigations of the effects of maternal separation on central nervous system development ^1^. On day 21, rats were weaned and housed 2 per cage under standard animal care conditions until adulthood (8-10 weeks old), at which time ventilatory measurements were performed.

**Whole-body plethysmography**

Ventilatory variables was measured in unrestrained, unanesthetized rats using a whole-body, flow-through plethysmograph (PLY3223 Buxco Electronics, Sharon, CT - USA). These experiments were first performed on pre-pubertal males and females aged between 14 and 15 days of age. Briefly, the system consists a Plexiglas experimental chamber of either 400 ml (pups) or 2L (adults). The system was calibrated by injecting a known volume (0.5ml or 5 ml) into the chamber with a glass syringe at a rate corresponding to the airflow range typically generated by the rat. Fresh air (baseline) or a hypercapnic gas mixture was delivered into the experimental chamber at a constant rate with a bias flow regulator (PLY1020; Buxco Electronics, Sharon, CT). Room air or the CO_2_ enriched gas mixture were delivered to the experimental chamber at a constant rate (pups: 380 – 400 ml/min; adults: 1.3 to 1.6 L/min). Barometric pressure, chamber temperature (T_C_), humidity and the rectal temperature of the animals (T_B_) were measured at the beginning and the end of each experiment to express the tidal volume (V_T_; and thus minute ventilation, V. e) in ml BTPS ^2^. Composition of the gas mixtures flowing in and out of the chamber was analyzed with an oxygen analyzer (model S-3A, Ametek, Pittsburgh, PA) for subsequent calculation of oxygen consumption (V. O_2_) in an open system ^3^.

*Experimental protocol*: 45 to 60 minutes before start the recordings, adult females were injected (i.p.) with the ORX_1_ receptor antagonist (SB334867; 15mg/kg; Sigma-Aldrich, Canada) or vehicle (50mM HP-β-CD - 10% + DMSO – 2%). The rat was then placed into the chamber and allowed to acclimatize before the measurements. Baseline (normocapnia) recordings were made when the animal was quiet and the ventilatory variables were stable. All measurements were performed between 13:00 to 15:00 to minimize changes in endocrine and respiratory activity associated with the circadian rhythm ^4^. The baseline values obtained were representative of the data recorded over the preceding 10 min. A gas mixture of 5% CO_2_ in air was then delivered to the chamber for 10 min. The CO_2_ level was chosen because similar levels are used in the clinic to evaluate PD patients ^5^. At the end of hypercapnia, the recording chamber was opened for a final body temperature measurement. Thirty minutes later, the rat was deeply anesthetized with a ketamine (Pfeizer, Kirkland, QC, Canada; 80 mg/kg) and xylazine (Bimeda, Cambridge, ON, Canada; 10 mg/ kg) and vaginal smears were performed according to the criteria of Goldman et al ^6^ to identify the phase of the estrous cycle.

Data analysis: Baseline measurements of ventilatory variables were obtained by averaging the last 10 min of stable recording. At the end of the hypercapnic exposure (5% CO_2_) a 5 min average was taken for each variable.

**Blood sampling and brain harvesting**

Blood and brain tissue samples were obtained either “at rest” (after 30 min of acclimation to the plethysmography chamber while breathing room air), or 30 min after exposure to 5% CO_2_). Once the rat was deeply anesthetised, blood samples were withdrawn from the left ventricle; samples were placed in a serum-gel clotting activator Microtube (Sarstedt AG & Co., Nümbrecht Germany). After centrifugation (12.000 rpm at 4°C for 12 min), blood serum was collected and placed in a −80°C freezer until assayed.

Brain tissue was then harvested in one of two ways. Fresh tissue was harvested for quantification of ORX_A_ in hypothalamic extracts. Dissection of this brain area was performed according to standard methods and landmarks ^7, 8^. The hypothalamic area was collected from the pre-optic area to the mid-brain boundary using the anterior commissure as the dorsal boundary. The brain samples were immediately frozen in liquid nitrogen and then kept at -80^o^C until experimentation. For immunohistochemistry, intra-cardiac perfusion was performed with 0.9% saline followed by 4% paraformaldehyde (PFA) in 0.1M sodium tetraborate buffer (PFA/borax; pH 9.5 at 4^o^C). Brains were removed from the skull, post-fixed for 24 h in 4% PFA/borax and then placed in 20% sucrose - 4% paraformaldehyde solution for 48 h at 4^o^C. Frozen brains were mounted on a microtome and the regions of interest were cut in 40 μm coronal sections. Slices were collected in a cold cryoprotectant solution (0.05M sodium phosphate buffer, 30% ethylene glycol and 20% glycerol) and stored at −20^o^C.

**Biochemical assays**

17β-estradiol (total E_2_) was measured using ELISA assay kits (Cayman Chemical Company; Ann Arbor, MI, USA). Assay was performed according to the manufacturer’s instructions and samples were read on a microplate spectrophotometer (Quant; Bio-Tek Instruments Inc., Winooski, VT, USA). According to the manufacturer, the sensitivity of the kits are 20 pg/ml; E_2_ concentrations were calculated from the four-parameter logistic standard curve using Sigma-Plot 12.3 (Systat Software, San Jose CA, USA).

Peptide extraction and orexin-A quantification. Tissue samples from the hypothalamus were processed according to Feng et al. ^7^. Briefly, 0.1 M acetic acid with a volume equal to 10 times the tissue weight was added to each tube. The microtubes were then moved to a boiling water bath for 10 min. The samples were then cooled and homogenized on ice. Additional 30 min were given to samples on ice before the microtubes were centrifuged for 15 min at 12000 rpm. The remaining supernatants (containing total peptides) were separated: 50 μl for assaying the total proteins then we kept the rest for orexin measure. Subsequently samples were stored at −80°C until quantification by commercially available enzyme immunoassay kit for orexin-A (Phoenix Pharmaceuticals Inc; Burlingame, CA; USA); the kit’s sensitivity is 0.22 ng/ml. Quantification was performed according to the manufacturer’s protocol using the same microplate reader and calculation method as E_2_.

***c-Fos* / orexin-A immunohistochemistry**

*c-Fos* protein immunolabeling was used as a functional marker of neuronal activation during normocapnia in the dorsomedial and lateral hypothalamus (DMH and LH, respectively) and perifornical area (PeF) (bregma -2.56 to -3.14 mm; ^8^). ORX_A_ co-labeling was performed to confirm cell phenotype. Brain sections containing DMH, LH, and PeF from NMS and CTRL were washed in PBS, quenched with 0.1% NaBH_4_ for 30 min in darkness. After PBS washes, sections were incubated for 2h at room temperature with blocking solution (0.3% Triton-X + 1% BSA + 5% Normal Donkey Serum). A cocktail of primary antibodies Orexin A (goat anti-orexin A ; Santa Cruz Biotechnologies SC 8070; 1:500)  and *c-Fos* (rabbit anti-human *c-Fos* polyclonal affinity-purified antibody; Calbiochem PC38, Ab-5; 1:1000) were diluted in the blocking solution and incubated for 1h at room temperature then at 4° overnight. Next morning, sections were incubated in the same solution for 1h at room temperature then were extensively washed with PBS. A cocktail of secondary antibodies donkey anti-rabbit IgG Alexa 555 (#A31572, Life Technology; 1:500) and donkey anti-goat IgG Alexa 488 (#A11055, Life Technology; 1:1000) were diluted in 0.3% Triton-X/PBS and incubated for 2h at room temperature. Sections were mounted on slides in cold PBS. Once dried, slides were cover-slipped with an aqueous mounting media (Fluoromount, Southern Biotech #0100-01).

Data analysis: Four sections per animal were analyzed for a total of six animals per condition (CTRL or NMS). Neuroanatomical boundaries of hypothalamus were identified using the atlas and the distribution of positive neurons for orexin A. Quantitative analyses of *c-Fos* and orexin A were collected on a Carl Zeiss Imager M2 microscope (Zeiss Microscopy, Germany) equipped with a camera Axiocam MRm (Zeiss Microscopy, Germany). Photographies for each fluorochrome were taken by using the software Zen 2 (Blue Edition, 2012, Zeiss Microscopy, Germany) and the tiles option were used to reconstruct the entire hypothalamus on each brain section. The photography were adjusted for brightness and contrast in Adobe Photoshop 13.0 (San Jose, CA) and the co-localization of each fluorochrome was quantified with ImageJ (NIH - National Institutes of Health, Bethesda, Maryland, USA, http://imagej.nih.gov/ij/, 1997-2016).

**Whole cell recording of orexin neurons**

*Identification of orexin neurons with an adeno-associated virus (AAV)* The hypothalamic region that contains ORX producing neurons can easily be located visually but unlike other groups of cells producing neuroactive substances (e.g. noradrenaline), ORX neurons do not form a dense population within a clearly delineated structure. To ensure clear identification of ORX-producing neurons prior to recording, 4 weeks old rats were injected with an AAV construct that expresses a green fluorescent protein (GFP) under the control of an ORX promoter. During stereotaxic surgery, rats received unilateral injection (1µl) of the ORX:GFP virus near ORX neurons (coordinates from Bregma: RC: -2.6 mm ; ML: 1.2 mm ; DV: -9.0 mm). Rats recovered 4 weeks. Comparison of GFP *versus* immunohistochemical labeling of ORX_A_ – positive cells confirmed the efficiency and specificity of the AAV.

*Hypothalamic slice preparation* Following isoflurane anesthesia, rats were decapitated and a vaginal smear was performed to determine the estrus cycle phase. The brain was carefully removed from the skull and immersed in ice-cold sucrose solution previously equilibrated with 95% O_2_ - 5% CO_2_ containing (in mM): NaCl 87; NaHCO_3_ 25; KCl 2.5; NaH_2_PO_4_ 1.25; MgCl_2_ 7; CaCl_2_ 0.5; glucose 25, and sucrose 75. The hypothalamus was dissected and frontal slices (300 µm) containing ORX neurons were cut with a vibratome (Leica Model VT1000S, Nussloch, Germany). Slices were then placed in oxygenated sucrose solution at 32°C for at least 30 min and then kept at room temperature until they were used for experiments. All recordings were performed using aCSF containing: NaCl 130; NaHCO_3_ 20; KCl 5; MgSO_4_ 1.3; CaCl_2_ 2.4, KH_2_PO_4_ 1.25, and glucose 10 (95% O_2_ - 5% CO_2_; pH = 7.4).

*Electrophysiological recordings* Slices were transferred to a recording chamber, secured with a nylon mesh, and superfused with aCSF at a flow rate of 2 ml/min and maintained at 32°C (TC-324C Heater Controller; Warner Instruments, Hamden, CT). Neurons were visualized under an upright Olympus microscope using a 40X water-immersion objective, equipped with differential interface contrast and an infrared-sensitive camera (Olympus XM 10, Richmond Hill, ON). GFP-labeled cells were identified with fluorescence: U-RFL-T mercury lamp with TRITC filter (ex: 549 nm, em: 565 nm). Since the densest GFP labeling was observed in the perifornical area (PeF), whole-cell recordings of ORX neurons were mostly performed in this area to minimise data variability.

Gigaseal formation was performed under voltage-clamp configuration. Briefly, patch electrodes (4 – 6.5 MΩ) were fabricated from 1.12 mm inside diameter filamented borosilicate glass (World Precision Instruments, Sarasota, FL, USA) using a vertical puller (Narishige Model PC-10 vertical puller, Amityville, NY, USA) and filled with a solution containing (mM): K-gluconate 120; KCl 20; MgCl_2_ 2; EGTA 0.6; Mg_2_ATP 2; NaGTP 0.3; Hepes 10; phosphocreatine 7 (285-290 mOsm). Data were recorded using an Axopatch 200B amplifier (Molecular Devices, Sunnyvale, CA, USA), digitized with Axon Digidata 1550 (Molecular Devices), sampled at 10 kHz, filtered at 2 kHz and acquired using pCLAMP 10 software (ver.10.7, Molecular Devices).

Schöne et al have described two types main types of ORX cells in mice (D and H) ^9^. We first identified the type of cell by performing the hyperpolarizing test describe in their study. This analysis showed that 88% of the cells were “D” type. Due to the difficulty of obtaining “H” type cells in each phase of the estrus cycle these cells not considered in the study.

Series resistance was monitored by delivery of 5 mV step throughout the experiment, a variation in series resistance >20% between baseline and subsequent measurements were not analyzed. Resting membrane potential was measured less than 30 seconds after establishing a stable whole-cell configuration and prior to any manipulations.

*EPSC response to 17β-estradiol (E_2_)* Excitatory postsynaptic currents (EPSCs) were recorded for 10 min at -60 mV under voltage clamp. E_2_ (100 nM in 5% DMSO) was applied onto cells for 10 min and a recovery period of 20 min was applied (without E_2_). Stability of the recordings was confirmed by applying DMSO (5%) onto cells over the same period than E_2_ (time control).

*Electrophysiological analysis*  Analysis of electrophysiological data was performed offline using Clampfit software (ver. 10.7, Molecular Devices, Sunnyvale, CA, USA). Action potential properties were measured from the first spike as follows: threshold – before the action potential upstroke; amplitude – from threshold to peak; half-width – measured at half the calculated amplitude; and AHP fast/medium – from threshold to maximum hyperpolarization.

Automated analyses of sEPSC frequency and amplitude were performed on cell using the “ClampFit” module of the recording software. The detection threshold was set at three times the SD of the baseline noise (Clements and Bekkers, 1997) so that no EPSCs were under 6.2 pA were considered in the analysis. This approach avoids the detection of false positives. Baseline measurements were obtained by averaging the 10 min of recording.

**References**

1. Lehmann J, Feldon J. Long-term biobehavioral effects of maternal separation in the rat: consistent or confusing? *Rev Neurosci* 2000; **11**(4)**:** 383-408.

2. Drorbough JE, Fenn WO. A Barometric method for measuring ventilation in newborn infants. *Pediatrics* 1955; **16:** 81-86.

3. Mortola JP, Dotta A. Effects of hypoxia and ambient temperature on gaseous metabolism of newborn rats. *Am J Physiol* 1992; **263**(2 Pt 2)**:** R267-272.

4. Mortola JP, Seifert EL. Circadian patterns of breathing. *Respir Physiol Neurobiol* 2002; **131**(1-2)**:** 91-100.

5. Roberson-Nay R, Klein DF, Klein RG, Mannuzza S, Moulton JL, Guardino M *et al.* Carbon dioxide hypersensitivity in separation-anxious offspring of parents with panic disorder. *Biol Psychiatry* 2010; **67**(12)**:** 1171-1177.

6. Goldman JM, Murr AS, Cooper RL. The rodent estrous cycle: characterization of vaginal cytology and its utility in toxicological studies. *Birth Defects Res B Dev Reprod Toxicol* 2007; **80**(2)**:** 84-97.

7. Feng P, Vurbic D, Wu Z, Strohl KP. Brain orexins and wake regulation in rats exposed to maternal deprivation. *Brain Res* 2007; **1154**(0)**:** 163-172.

8. Paxinos G, Watson C. *The rat brain in stereotaxic coordinates*. 4th Edition edn. Academic Press: San Diego, 1998.

9. Schöne C, Venner A, Knowles D, Karnani MM, Burdakov D. Dichotomous cellular properties of mouse orexin/hypocretin neurons. *J Physiol* 2011; **589**(11)**:** 2767-2779.
