# Supplementary material for: Disruption of estradiol regulation of orexin neurons: a novel mechanism in excessive ventilatory response to CO2 inhalation in a female rat model of panic disorder
Source: Transl Psychiatry. 2020 Nov 10;10:394. doi: 10.1038/s41398-020-01076-x (PMC7656265; doi:10.1038/s41398-020-01076-x)
Supplement: Supplementary file 2 — Supplementary Table 1 [file 41398_2020_1076_MOESM2_ESM.docx]

|  | *Intact +*  *Vehicle* | | *Intact +*  *SB 334867* | | *Ovariectomy*  *+ vehicle* | | *Ovariectomy*  *+ estradiol* | | ANOVA | | |
| --- | --- | --- | --- | --- | --- | --- | --- | --- | --- | --- | --- |
|  | Control  (n = 62) | NMS  (n = 49) | Control  (n = 22) | NMS  (n = 24) | Control  (n = 17) | NMS  (n = 6) | Control  (n = 22) | NMS  (n = 14) | Stress  Effect | Treatment  Effect | Factorial  Interaction |
| Age (days) | 65.4  ± 0.5 | 64.6  ± 0.4 | 64.9  ± 0.8 | 65.3  ± 0.6 | 61.8*****  ± 1.1 | 58.8  ± 1.1 | 61.5*****  ± 0.7 | 60.6*****  ± 0.6 | *P* = 0.12  *F*_(1,208)_ = 2.42 | ***P* < 0.0001**  F_(3,208)_ = 0.16 | NS |
| Weight (g) | 285  ± 4 | 277  ± 4 | 281  ± 7 | 297*****  ± 6 | 318  ± 8***** | 317*****  ± 11 | 314*****  ± 7 | 298*****  ± 8 | *P* = 0.69  *F*_(1,208)_ = 0.16 | ***P* < 0.0001**  *F*_(3,208)_ = 11.3 | NS |
| Body temperature  (°C) | 37.5  ± 0.1 | 37.7  ± 0.08 | 37.9*****  ± 0.1 | 38.0  ± 0.1 | 37.1*****  ± 0.1 | 37.5  ± 0.3 | 37.0*****  ± 0.1 | 37.4  ± 0.2 | ***P* = 0.02**  *F*_(1,208)_ = 5.3 | ***P* < 0.0001**  *F*_(3,208)_ = 9.7 | NS |
| Breathing frequency  (breaths/min^-1^) | 87  ± 1 | 85  ± 2 | 82  ± 2 | 81  ± 2 | 96*****  ± 3 | 95*****  ± 3 | 93*****  ± 3 | 91*****  ± 3 | *P* = 0.38  *F*_(1,208)_ = 0.76 | ***P* < 0.0001**  *F*_(3,208)_ = 10.5 | NS |
| Tidal volume  (ml BTPS/100g) | 0.62  ± 0.02 | 0.67  ± 0.02 | 0.62  ± 0.02 | 0.66  ± 0.03 | 0.69  ± 0.05 | 0.58  ± 0.05 | 0.69  ± 0.04 | 0.67  ± 0.06 | *P* = 0.70  *F*_(1,208)_ = 0.15 | *P* = 0.67  *F*_(3,208)_ = 1.3 | NS |
| Minute ventilation  (ml BTPS/100g) | 58  ± 2 | 57 ± 2 | 51  ± 2 | 52  ± 2 | 65  ± 4 | 55  ± 5 | 64  ± 4 | 62  ± 6 | *P* = 0.22  *F*_(1,208)_ = 1.48 | ***P* = 0.008**  *F*_(3,208)_ = 4.06 | NS |
| Oxygen consumption  (ml STPD/min/100g) | 1.90  ± 0.06 | 1.84  ± 0.06 | 1.94  ± 0.07 | 1.90  ± 0.05 | 2.2*****  ± 0.1 | 2.6*****  ± 0.2 | 2.11  ± 0.08 | 2.2*****  ± 0.1 | *P* = 0.21  *F*_(1,195)_ = 1.59 | ***P* < 0.0001**  *F*_(3,195)_ = 10.13 | NS |
| Oxygen convective requirement ratio | 33  ± 2 | 33  ± 2 | 26  ± 1 | 29  ± 2 | 31  ± 3 | 22*****  ± 2 | 31  ± 2 | 29  ± 3 | *P* = 0.35  *F*_(1,195)_ = 0.89 | ***P* = 0.04**  *F*_(3,195)_ = 2.78 | NS |

**Table 1:** **Comparison of age, weight, ventilatory and metabolic values obtained during baseline.** The values are compared between female rats exposed to control or neonatal stress (NMS) protocol that received saline or SB334867. Data are reported as means ± SEM. ***** indicates a value significantly different from corresponding intact value at *p* < 0.05.
