# Supplementary material for: Disruption of estradiol regulation of orexin neurons: a novel mechanism in excessive ventilatory response to CO2 inhalation in a female rat model of panic disorder
Source: Transl Psychiatry. 2020 Nov 10;10:394. doi: 10.1038/s41398-020-01076-x (PMC7656265; doi:10.1038/s41398-020-01076-x)
Supplement: Supplementary file 3 — Supplementary Table 2 [file 41398_2020_1076_MOESM3_ESM.docx]

| ***Cell properties*** | **Control** | | | | **Neonatal maternal separation (NMS)** | | | | **ANOVA** | | |
| --- | --- | --- | --- | --- | --- | --- | --- | --- | --- | --- | --- |
|  | *Proestrus* | *Estrus* | *Metestrus* | *OVX* | *Proestrus* | *Estrus* | *Metestrus* | OVX | Stress effect | Phase effect | Factorial interaction |
| Cell capacitance (pF) | (N=5; n=9)  62 ± 8.7 | (N=4; n=8)  65 ± 8.5 | (N=7; n=11)  58 ± 6.8 | (N=7; n=9)  92 ± 3.0 | (N=5; n=13)  69 ± 7.5 | (N=4; n= 8)  64.7 ± 4.9 | (N=3; n=5)  55 ± 12.7 | (N=9; n=16)  68 ± 6.1 | *P* = 0.34  *F*_(1,71)_ = 0.94 | ***P* = 0.02**  *F*_(3,71)_ = 3.52 | NS |
| Membrane potential (mV) | -44.3 ± 0.8 | -41.9 ± 1.1 | -42.2 ± 1.2 | -44.4 ± 1.3 | -44.8 ± 1.2 | -44.5 ± 1.4 | -45.6 ± 1.8 | -42.0 ± 0.4**†** | *P* = 0.22  *F*_(1,71)_ = 1.56 | *P* = 0.53  *F*_(3,71)_ = 0.74 | ***P* = 0.05**  *F*_(3,71)_ = 2.69 |
| ***Spontaneous action potentials*** | | | | | | | | | | | |
| Frequency (Hz) | 5.0 ± 1.1 | 3.9 ± 0.8 | 4.4 ± 1.0 | 4.4 ± 0.9 | 4.0 ± 0.7 | 4.0 ± 0.9 | 4.0 ± 1.4 | 4.8 ± 0.6 | *P* = 0.73  *F*_(1,71)_ = 0.12 | *P* = 0.89  *F*_(3,71)_ = 0.21 | NS |
| Threshold (mV) | -28 ± 2.2 | -28 ± 2.4 | -26 ± 1.4 | -30 ± 1.6 | -30 ± 1.8 | -30 ± 1.8 | -27 ± 3.8 | -25 ± 1.2 | *P* = 0.97  *F*_(1,71)_ = 0.001 | *P* = 0.62  *F*_(3,71)_ = 0.59 | NS |
| Amplitude (mV) | 65 ± 3.1 | 71 ± 2.1 | 67 ± 3.6 | 69 ± 2.5 | 72 ± 3.1 | 71 ± 2.8 | 66 ± 7.1 | 63 ± 2.6 | *P* = 0.94  *F*_(1,71)_ = 0.006 | *P* = 0.47  *F*_(3,71)_ = 0.86 | NS |
| Half-width (ms) | 1.10 ± 0.06 | 1.05 ± 0.03 | 1.03 ± 0.07 | 1.15 ± 0.06 | 1.07 ± 0.06 | 1.13 ± 0.06 | 1.13 ± 0.18 | 1.20 ± 0.05 | *P* = 0.35  *F*_(1,71)_ = 0.87 | *P* = 0.84  *F*_(3,71)_ = 0.28 | NS |
| AHP amplitude (mV) | -28 ± 2.1 | -26 ± 2.3 | -24 ± 1.8 | -26 ± 0.9 | -24 ± 1.4 | -25 ± 1.2 | -28 ± 2.2 | -26 ± 1.1 | *P* = 0.75  *F*_(1,71)_ = 0.1 | *P* = 0.98  *F*_(3,71)_ = 0.06 | NS |
| ***Spontaneous EPSC*** | | | | | | | | | | | |
| Rise time (ms) | (N=3; n=5)  1.06 ± 0.14 | (N=5; n=8)  1.35 ± 0.22 | (N=7; n=11)  1.05 ± 0.09 | (N=5; n=6)  0.90 ± 0.20 | (N=5; n=11)  1.20 ± 0.18 | (N=4; n=4)  1.12 ± 0.04 | (N=3; n=4)  1.09 ± 0.13 | (N=9; n=13)  1.23 ± 0.08 | *P* = 0.55  *F*_(1,54)_ = 0.36 | *P* = 0.74 *F*_(3,54)_ = 0.41 | NS |
| Decay time (ms) | 2.05 ± 0.15 | 2.91 ± 0.46 | 2.73 ± 0.15 | 2.61 ± 0.33 | 3.17 ± 0.35 | 2.52 ± 0.21 | 2.64 ± 0.33 | 3.16 ± 0.15 | *P* = 0.21  *F*_(1,54)_ = 1.64 | *P* = 0.83 *F*_(3,54)_ = 0.29 | NS |

**Table 2:** **Effects of neonatal stress on the basic properties of orexin neurons of the perifornical/dorsomedial area of the hypothalamus.** The values are compared between female rats across the estrus cycle and 14 days following ovariectomy (OVX). Data are reported as means ± SEM. Values in bracket in indicate the number of animals (N) and the number of cells (n) in each group. ***** indicates a value significantly different from corresponding proestrus value at *P* < 0.05. **†** indicates a value significantly different from corresponding control value at *P* < 0.05.
