# Supplementary figures and images for: Disruption of estradiol regulation of orexin neurons: a novel mechanism in excessive ventilatory response to CO2 inhalation in a female rat model of panic disorder
Source: Transl Psychiatry. 2020 Nov 10;10:394. doi: 10.1038/s41398-020-01076-x (PMC7656265; doi:10.1038/s41398-020-01076-x)

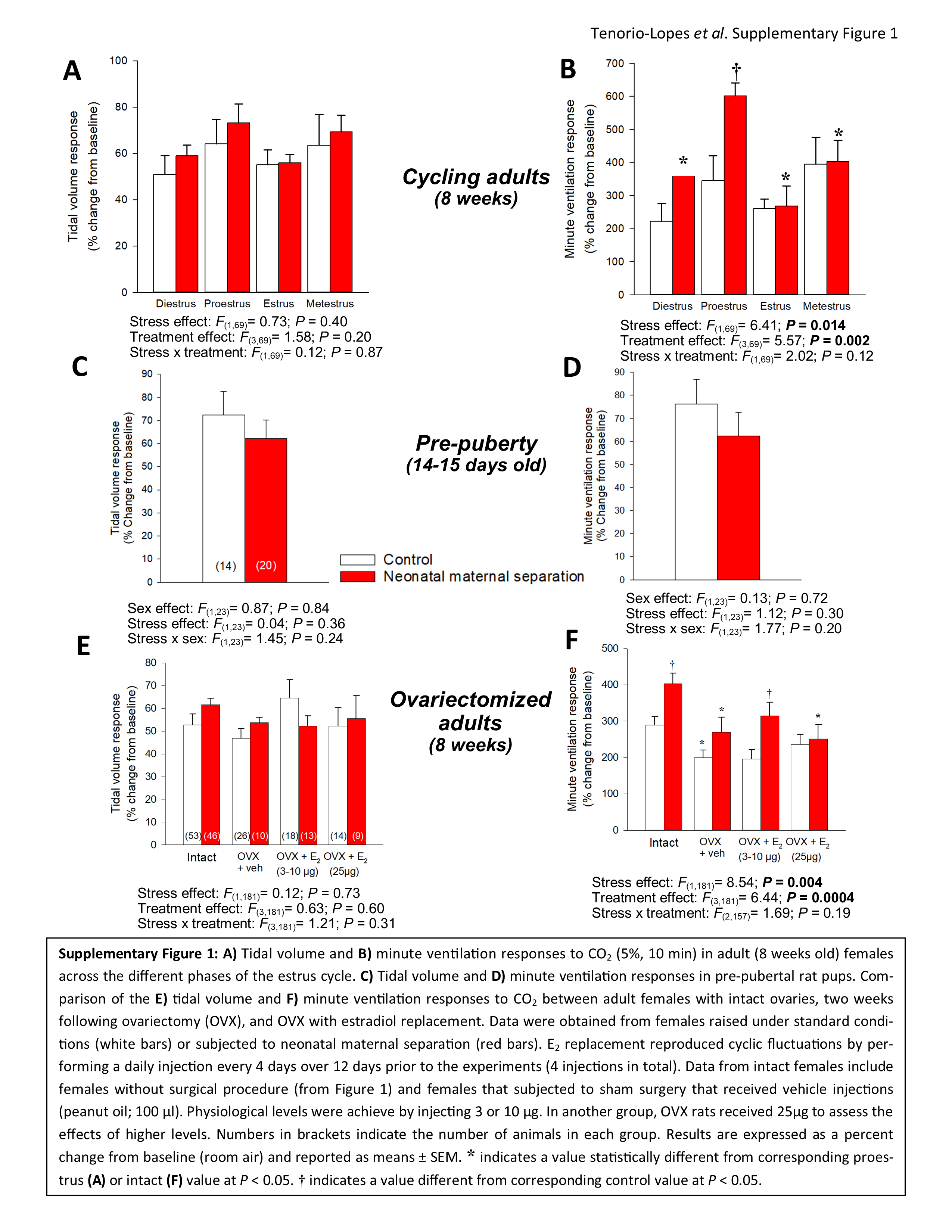

Supplement: Supplementary file 4 — Supplementary Figure 1 [file 41398_2020_1076_MOESM4_ESM.tif]

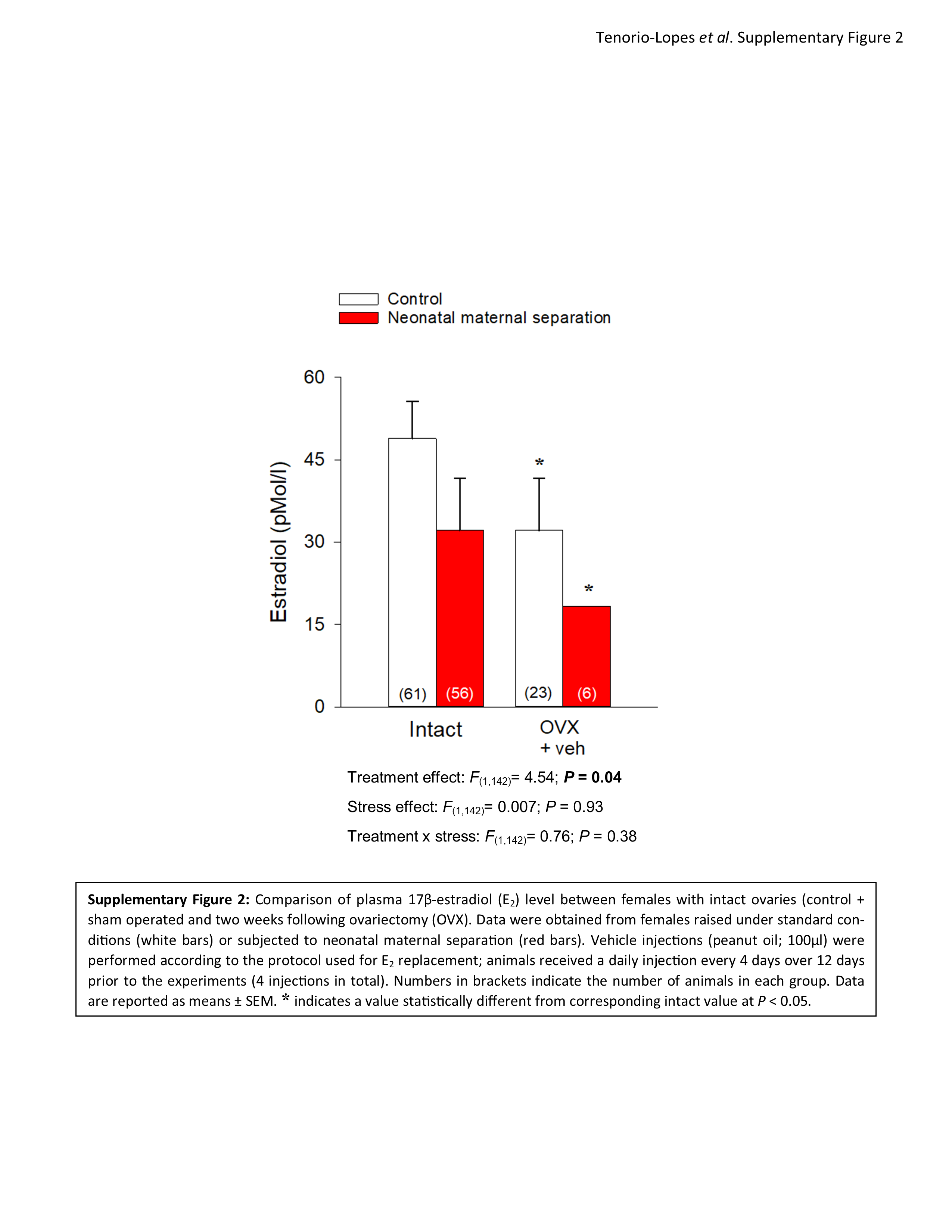

Supplement: Supplementary file 5 — Supplementary Figure 2 [file 41398_2020_1076_MOESM5_ESM.tif]

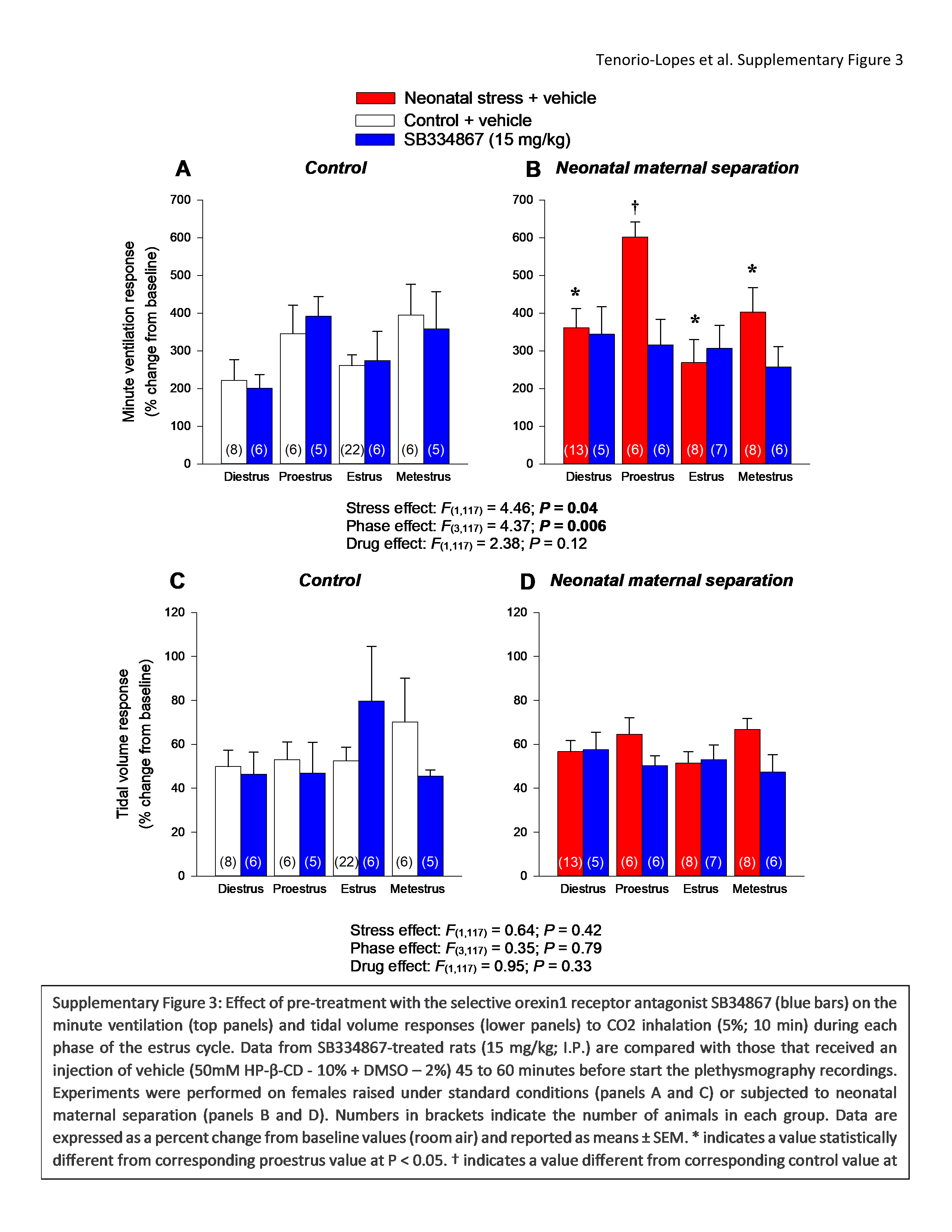

Supplement: Supplementary file 6 — Supplementary Figure 3 [file 41398_2020_1076_MOESM6_ESM.tif]
